# Homogenous multifunctional microspheres induce ferroptosis to promote the anti-hepatocarcinoma effect of chemoembolization

**DOI:** 10.1186/s12951-022-01385-x

**Published:** 2022-04-02

**Authors:** Minjiang Chen, Jie Li, Gaofeng Shu, Lin Shen, Enqi Qiao, Nannan Zhang, Shiji Fang, Xiaoxiao Chen, Zhongwei Zhao, Jianfei Tu, Jingjing Song, Yongzhong Du, Jiansong Ji

**Affiliations:** 1 Institute of Pharmaceutics, College of Pharmaceutical Sciences, Zhejiang University, Hangzhou, 310058 China; 2 Key Laboratory of Imaging Diagnosis and Minimally Invasive Intervention Research, Lishui Hospital of Zhejiang University, The Fifth Affiliated Hospital of Wenzhou Medical University, Lishui, 323000 China; 3 Department of Medical Imaging, Ningbo Women & Childen’s Hospital, Ningbo, 315012 China

**Keywords:** Fe_3_O_4_ nanoparticles, Ferroptosis, Homogenous drug-loaded microspheres, Transcatheter arterial chemoembolization, Hepatocellular carcinoma

## Abstract

Transcatheter arterial chemoembolization (TACE) is one of the main palliative therapies for advanced hepatocellular carcinoma (HCC), which is also regarded as a promising therapeutic strategy for cancer treatment. However, drug-loaded microspheres (DLMs), as commonly used clinical chemoembolization drugs, still have the problems of uneven particle size and unstable therapeutic efficacy. Herein, gelatin was used as the wall material of the microspheres, and homogenous gelatin microspheres co-loaded with adriamycin and Fe_3_O_4_ nanoparticles (ADM/Fe_3_O_4_-MS) were further prepared by a high-voltage electrospray technology. The introduction of Fe_3_O_4_ nanoparticles into DLMs not only provided excellent T2-weighted magnetic resonance imaging (MRI) properties, but also improved the anti-tumor effectiveness under microwave-induced hyperthermia. The results showed that ADM/Fe_3_O_4_-MS plus microwave irradiation had significantly better antitumor efficacy than the other types of microspheres at both cell and animal levels. Our study further confirmed that ferroptosis was involved in the anti-tumor process of ADM/Fe_3_O_4_-MS plus microwave irradiation, and ferroptosis marker GPX4 was significantly decreased and ACSL4 was significantly increased, and ferroptosis inhibitors could reverse the tumor cell killing effect caused by ADM/Fe_3_O_4_-MS to a certain extent. Our results confirmed that microwave mediated hyperthermia could amplify the antitumor efficacy of ADM/Fe_3_O_4_-MS by activating ferroptosis and the introduction of Fe_3_O_4_ nanoparticles can significantly improve TACE for HCC. This study confirmed that it was feasible to use uniform-sized gelatin microspheres co-loaded with Fe_3_O_4_ nanoparticles and adriamycin to enhance the efficacy of TACE for HCC.

**Graphical Abstract:**

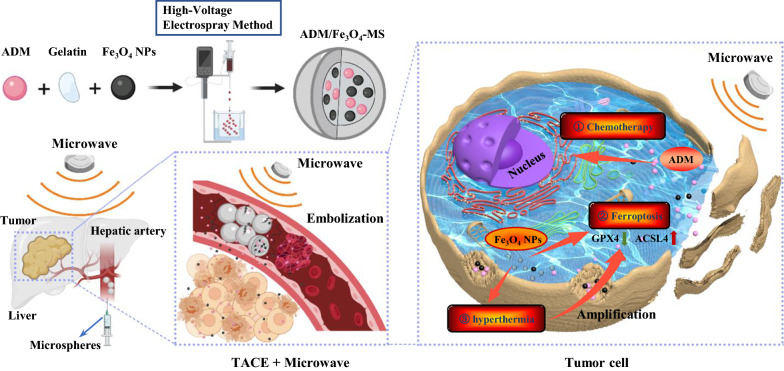

**Supplementary Information:**

The online version contains supplementary material available at 10.1186/s12951-022-01385-x.

## Introduction

Hepatocellular carcinoma (HCC) remains a global health challenge and is also one of the major contributors to the worldwide cancer burden, what’s more serious is that its incidence and mortality are rapidly rising around the world [1, 2]. HCC is an aggressive and highly malignant liver tumor with a poor 5-year survival rate, which is largely due to the fact that most patients are clinically diagnosed in the advanced stages [3]. Interventional comprehensive therapy represented by transcatheter arterial chemoembolization (TACE) plays an important role in the treatment of patients with advanced HCC [4], and a growing number of studies have pointed out that TACE has significant benefits for these patients [5, 6]. However, as a palliative therapy, TACE still has the problem of poor tumor response and residual tumor tissue prone to progression and recurrence. At present, how to further improve the efficacy of TACE is still a key issue needed to be solved urgently to maximize the benefits of patients.

An increasing number of evidences have confirmed that the tumor hypoxia microenvironment caused by TACE reduces the sensitivity of chemotherapy, which is also an important factor for the unstable therapeutic effect of TACE [7, 8]. Hypoxia is one of the important signs of tumors, which can induce changes in gene expression and subsequent proteomic levels, and further affect cellular and physiological functions, thereby affecting the prognosis [9, 10]. What’s more, the TACE aggravates the intratumoral hypoxia state and oxygen gradient distribution, which also could contribute to the plasticity and heterogeneity of tumors and promoting the chemotherapy resistance of tumors [11, 12]. The reduction of sensitivity to chemotherapy is an important way that hypoxia affects the efficacy of chemoembolization [13, 14], which also provides an important strategy for clinical practice.

Extensive researches have shown that hyperthermia is a potent sensitizer for tumor chemotherapy resistance, which can also greatly enhance the effectiveness of chemotherapy [15, 16]. Hyperthermia has also been confirmed to improve the anti-tumor efficacy of chemotherapeutic drugs by increasing the delivery efficiency and distribution [17]. Our previous studies have also shown that radiofrequency hyperthermia can improve the distribution of chemotherapy drugs in the tumor and enhance the killing effect of tumor cells [18]. Therefore, if hyperthermia can be introduced into the existing chemoembolization treatment system, it will significantly improve the efficacy of TACE treatment. Drug-loaded microspheres (DLMs), as a commonly used clinical chemoembolization drug for HCC, have been proved to be superior to lipiodol-based chemoembolization mode [19, 20]. Herein, gelatin would be used in the preparation of DLMs in the present study, which is a commonly used embolization material in clinical practice, and its safety and stability have been fully confirmed [21, 22]. In order to give the gelatin microspheres the function of hyperthermia, we further introduced superparamagnetic iron oxide nanoparticles (SPIONs) into the DLMs system on the basis of loading chemotherapy drugs. SPIONs, as the thermal seeds, can absorb microwave and convert the radiation energy to heat, and enable us to accumulate more thermal energy into the tumor area to alter the tumor microenvironment by this way [23, 24]. The heat generation principle of SPIONs is to absorb microwave energy through unpaired electrons (Fe^2+^, Fe^3+^), and the excited electrons return into the ground state by releasing phonons, thus convert the microwave energy into heat energy [25]. The thermal conversion ability of SPIONs will give the microspheres the ability to hyperthermia therapy.

The goal of hyperthermia is to raise temperature in tumors to render the cells more sensitive to chemotherapy caused by DLMs. The sensitization occurs mostly via increased blood perfusion, increased oxygenation, inhibition of chemotherapy-induced DNA damage repair, and boosting local/systemic immune response according to previous researches [26, 27]. Interestingly, SPIONs are widely used for biomedical applications, and shows excellent performance as contrast agent for magnetic resonance imaging (MRI) and often used as the T2 contrast agents [28]. The introduction of SPIONs will also realize the visualization of DLMs under MRI, which can monitor the movement of microspheres in vivo and achieve precise tumor chemoembolization. What’s more, SPIONs could release Fe^2+^ and Fe^3+^ in the tumor and further induce ferroptosis of tumor cells, which is a regulated process of cell death caused by iron-dependent accumulation of lipid hydroperoxides (LPO) [29]. Previous studies have confirmed that SPIONs can induce Fenton-Reaction and cause ferroptosis, and further enhance tumor immunotherapy, which has been shown to be feasible in the treatment of a variety of tumors [30–32]. This also provides an important basis for the present study to improve the efficacy of chemoembolization through the novel DLMs loaded with SPIONs.

**Scheme 1:**
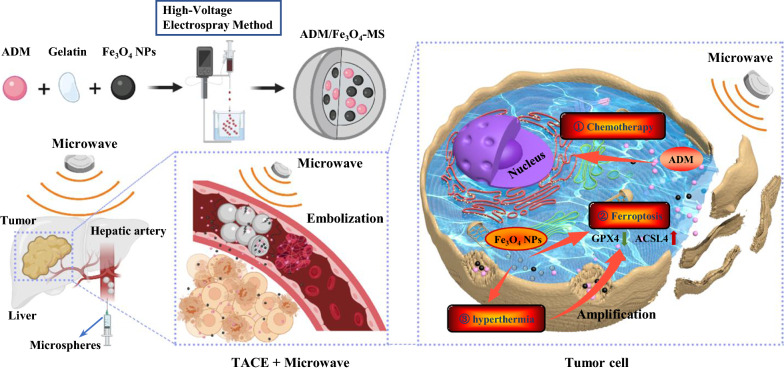
Schematic diagram of the preparation process of ADM/Fe_3_O_4_-MS and its TACE treatment mode

In our present study, the high-voltage electrospray method would be applied to prepare a novel gelatin microsphere dual-loaded with adriamycin (ADM) and Fe_3_O_4_ nanoparticles (ADM/Fe_3_O_4_-MS) with controllable particle size (Scheme 1). Based on Fe_3_O_4_ nanoparticles, the above microspheres not only realize visual monitoring under MRI, but also can effectively enhance the tumor killing effect of chemotherapeutics in situ through microwave-mediated hyperthermia therapy. Meanwhile, the high-voltage electrospray method can realize the homogenization of particle size of microspheres and effectively avoid ectopic embolization of microspheres. This study will comprehensively explore the anti-tumor efficacy and mechanism of the microspheres in vitro and in vivo, and provide a basis for the introduction of SPIONs into the clinical chemoembolization treatment system.

## Materials and methods

### Material and animals

Gelatin was purchased from Sigma-Aldrich (St. Louis, MO, USA). Glutaraldehyde (25% aqueous solution), Adriamycin hydrochloride, Fe_3_O_4_ nanoparticles (15–30 nm, 5 mg/mL in H_2_O) and Span-80 were obtained from Aladdin (Shanghai, China). Liquid paraffin, isopropanol and petroleum ether were purchased from Sinopharm (Beijing, China). Phosphate buffer saline (PBS) was purchased from Solarbio Technology (Beijing, China). All reagents are analytically pure.

The human HCC cell line LM3 was purchased from the American Type Culture Collection (ATCC; Manassas, VA, USA), and the LM3 hepatoma cells were cultured in high glucose Dulbecco’s modified Eagle medium (DMEM) (Gibco BRL, USA) containing 1% antibiotic–antimycotic, and 10% fetal bovine serum (Gibco BRL, USA) in a CO_2_ incubator at 37 °C.

A total of 31 male New Zealand White rabbits (weighing 2–2.5 kg) were obtained from Kangda Rabbit Co., Ltd. (Qingdao, China), and all rabbits were bred in the Laboratory Animal Center of our institution and had free access to standard diets and water during the experimental period. All the operations performed on the rabbits were in accordance with the national regulations and approved by the Zhejiang University Institutional Animal Care and Use Committee (No. 14650).

### Preparation of gelatin microspheres dual-loaded adriamycin (ADM) and Fe_3_O_4_ nanoparticles (ADM/Fe_3_O_4_-MS)

**Fig. 1:**
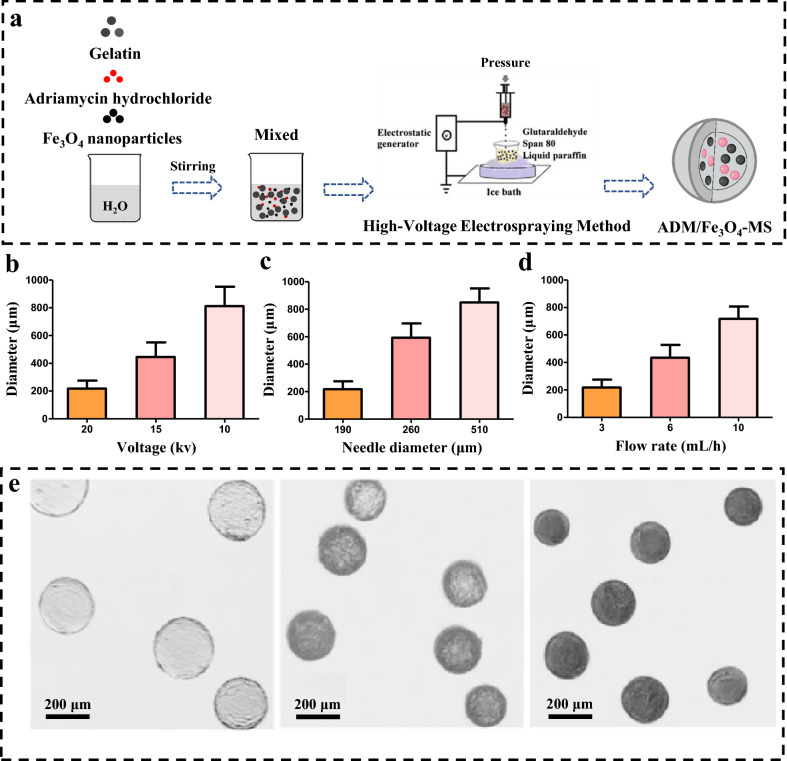
Study on preparation conditions of homogenous ADM/Fe_3_O_4_-MS. **a** Schematic diagram of the preparation process of ADM/Fe_3_O_4_-MS. **b** Particle sizes of ADM/Fe_3_O_4_-MS prepared under different voltage. **c** Particle sizes of ADM/Fe_3_O_4_-MS prepared with different needle diameters. **d** Effect of flow rates on particle size of ADM/Fe_3_O_4_-MS. **e** Representative microscope images of MS, ADM-MS and ADM/Fe_3_O_4_-MS

In our present work, a high-voltage electrospray technique was applied to prepare ADM/Fe_3_O_4_-MS. Briefly, 200 mg gelatin, 10 mg adriamycin hydrochloride and 3 mL Fe_3_O_4_ nanoparticles (5 mg/mL, 15–30 nm) were dispersed into 7 mL deionized water by probe sonication (JY-II DN, Ningbo Xinzhi Biological Technology Co., Ltd., China) for 10 min (600 W, 2 s-active and 3 s-inactive). The suspension was loaded into a syringe equipped with a blunt stainless-steel needle, which was vertical to the ground. Then, the suspension was pressed out by a syringe pump (WZ-50C6, Zhejiang Smith Medical Instrument Co., Ltd. China) at a constant flow rate. A collecting bath was located at 10 cm downward from the needle, which containing 200 μL of glutaraldehyde solution (25%), 200 μL of span 80 and 15 mL of paraffin liquid as the oil phase. The collecting solution was churned mildly under ice bath. Two high voltage power supplies (Type GF-II, Suzhou City Paint Engineering Co., Ltd., China) were connected to the needle and collecting bath respectively, and the potential difference between the needle and the collecting bath was setting as 20 kV. The diameter and morphology of the microspheres would be adjusted by regulating the flow rate, applied voltage and diameter of the needle. After electrospraying, the microspheres were filtered and washed with isopropanol and petroleum ether for several times. To next, the collected microspheres were dried naturally and protected from light. The schematic diagram of ADM/Fe_3_O_4_-MS preparation is shown in Fig. 1a. The gelatin microspheres (MS) and gelatin microspheres loaded with Adriamycin (ADM-MS) were prepared in the same way.

Meanwhile, the effects of voltage, needle diameter and injection velocity on the morphology and particle size of ADM/Fe_3_O_4_-MS were further investigated.

### Evaluation of physicochemical properties of ADM/Fe_3_O_4_-MS

The morphology of the prepared microspheres was observed by inverted microscopy (Axio Observer A1, Carl Zeiss AG, Germany). The average diameter of the different microspheres was measured and calculated. The drug entrapment efficiency (EE) and loading capacity (LC) of the microspheres were further measured by extraction method. Briefly, 10 mg ADM-MS or ADM/Fe_3_O_4_-MS was weighed and the adriamycin was extracted from the microspheres in 10 mL DMSO using probe sonication for 2 h (600 W, 2 s-active and 3 s-inactive). The extracted adriamycin was analyzed using UV–Visible spectrophotometer (wavelength: 480 nm) [33] and assessed by visual inspection of the digest solutions. The contents of Fe_3_O_4_ were analyzed by inductively coupled plasma mass spectrometry (ICP-MS) [34]. All the experiments were repeated three times. The drug loading capacity and the amount of adriamycin encapsulated in the microspheres were calculated from the following equations:

1. $${\text{Loading Capacity }}\left( \% \right) = \left( {{\text{C}} \times {10}\,\,{\text{mL}} \times {{{10}^{ - 3} } \mathord{\left/ {\vphantom {{{10}^{ - 3} } {10\,{\text{mg}}}}} \right. \kern-\nulldelimiterspace} {10\,{\text{mg}}}}} \right) \times 100\%$$
2. $${\text{Entrapment Efficiency }}\left( \% \right) = {{\left( {{\text{C}} \times {10}\,\,{\text{mL}} \times {10}^{ - 3} } \right)} \mathord{\left/ {\vphantom {{\left( {{\text{C}} \times {10}\,\,{\text{mL}} \times {10}^{ - 3} } \right)} {{\text{Q}}_{{\text{t}}} }}} \right. \kern-\nulldelimiterspace} {{\text{Q}}_{{\text{t}}} }} \times 100\%$$

The concentration of adriamycin in DMSO was calculated by the adriamycin calibration with a concentration range from 8 to 24 μg/mL and denoted as C. Where Q_t_ is the quantity of drug added for encapsulation.

### In vitro drug release behavior of ADM/Fe_3_O_4_-MS and ADM-MS

We further explored the in vitro release behavior of adriamycin from ADM/Fe_3_O_4_-MS and ADM/MS. Firstly, 10 mg ADM/Fe_3_O_4_-MS and ADM/MS were individually added into the centrifuge tubes with 10 ml PBS buffer (pH = 7.4). Then, the centrifuge tubes were horizontally shaken with a rate of 45 rpm in an incubator at 37 °C. 5 mL of sample was withdrawn at 0, 1, 2, 4, 6, 10, 14, 18 and 22 days, and the corresponding same volume of PBS buffer was replaced. The concentration of ADM in the sample was also detected by UV–Visible spectrophotometer. The measurements were repeated three times. To investigate the effect of microwave on the release of ADM, the same amounts of samples were intervened with microwave for 30 min (25 W) at the selected time interval (0, 1, 2, 4, 6 days). The following procedures were the same as the one described above.

### Evaluation of in vitro MRI performance and superparamagnetic properties of ADM/Fe_3_O_4_-MS

ADM/Fe_3_O_4_-MS loaded with Fe_3_O_4_ nanoparticles, as the magnetic microspheres, we further confirmed its in vitro MRI performance and superparamagnetism. For MRI performance, 0, 1.5, 4.5 and 10.5 mg ADM/Fe_3_O_4_-MS, 10.5 mg MS and 10.5 mg ADM-MS were placed in a centrifuge tube containing 10% gelatin solution, which was used for the fixation of microspheres. All the microspheres were scanned by T2 weighted images, and the parameters of the scanned sequence were as follows: TR = 2000 ms, TE = 90 ms, reversal angle = 90°, slice thickness = 3.0 mm. Meanwhile, the T2-weighted relaxation value of MS, ADM-MS and ADM/Fe_3_O_4_-MS with different contents were also detected. For the superparamagnetic properties, 15 mg of ADM/Fe_3_O_4_-MS were weighed, and its superparamagnetism was determined by vibrating sample magnetometer.

### In vitro* thermal *efficiency* of ADM/Fe*_*3*_*O*_*4*_*-MS*

The thermal efficiency of the ADM/Fe_3_O_4_-MS was studied by monitoring the temperature change with the intervention of microwaves (MTC-3, Shanghai Viscon Medical Eletronics CO., Itd, China). 10 mg ADM/Fe_3_O_4_-MS were put into the test tube and 1.5 mL pure water was added. After microwave treatment (25 W, 1 h), the temperature of suspension was recorded by a thermometer at 0, 5, 10, 15, 20, 25, 30, 45 and 60 min, respectively. The pure water was used as the control group.

### In vitro cytotoxicity of ADM/Fe_3_O_4_-MS

In order to clarify the in vitro cytotoxicity of different microspheres in this study, cell viability was firstly determined by MTT assay. Briefly, the LM3 cells were placed in a 12-well transwell chamber (3.0 μm pore polycarbonate membrane transwell inserts, Corning, Inc., Lowell, MA) with a density of 5 × 10^4^ cells per chamber and incubated overnight. Then, 10 mg/mL MS, ADM-MS and ADM/Fe_3_O_4_-MS were added into corresponding transwell chambers and incubated for 48 h, and the cells without exposure to the microspheres were used as control. After that, 200 μL of a 3-(4, 5-dimethylthiazol-2-yl)-2, 5-diphenyltetrazolium bromide (MTT) solution (5 mg/mL) was added into each well for an additional 4 h incubation at 37 °C. Subsequently, the culture medium of each chamber was removed and 1 mL DMSO was added and shook for 15 min, and the absorption was measured by a microplate reader at 570 nm. Meanwhile, the microwave intervention was further carried out to certify whether it affects the survival of cells. For the microwave treated groups, the microwave (25 W, 30 min) was applied after adding the microspheres 12 h. The measurements were repeated in three times. The cell viability (V) was calculated by using the following formula:

1. $${\text{V}} = {{{\text{A}}_{{\text{m}}} } \mathord{\left/ {\vphantom {{{\text{A}}_{{\text{m}}} } {{\text{A}}_{{\text{c}}} }}} \right. \kern-\nulldelimiterspace} {{\text{A}}_{{\text{c}}} }} \times 100\% ,$$

Where A_m_ is the absorption value of the different microspheres and A_c_ is the control group.

To further confirm the effect of ADM/Fe_3_O_4_-MS on cell proliferation, the crystal violet staining assay was performed as a special stain [35]. 1 × 10^3^ per well of the LM3 cells were placed in the 6-well transwell chambers and incubated overnight, and then exposed to the MS, ADM-MS and ADM/Fe_3_O_4_-MS for further 24 h, and the cells without microsphere treatment were used as the control group. Then, the different microspheres were discarded from all the chambers, and replaced with fresh medium, and continued to incubate for 14 days for crystal violet staining to observe the cell proliferation by the inverted fluorescence microscope (Leica DMi8; Leica Microsystems, German). For the effects of microwave on the cell proliferation, we also treated different groups of cells with microwave for 30 min (25 W) after the addition of microspheres, and then the crystal violet staining was also performed after 14 days of incubation.

### Evaluation of in vitro therapeutic effect of ADM/Fe_3_O_4_-MS

In addition, the apoptotic or necrotic LM3 cells were measured by the flow cytometry and Live/Dead fluorescent staining assay to determine the in vitro therapeutic effect of ADM/Fe_3_O_4_-MS. Briefly, the tumor cell apoptosis was performed by Annexin V-FITC apoptosis assay kit (Shanghai univ biotechnology CO.,Itd, China) according to the protocol provided by the manufacturer. Briefly, LM3 cells were placed in the 6-well transwell chamber at a density of 5 × 10^4^ cells per well and incubated overnight, and then 10 mg/mL MS, ADM-MS and ADM/Fe_3_O_4_-MS were used for 24 h intervention. The LM3 cells in the microwave treatment group were also treated for 30 min after 12 h addition of different microspheres. After that, the LM3 cells treated with different microspheres were collected and washed with PBS, and the Annexin V and propidium iodide staining were performed, which were further analyzed by flow cytometry. For the LM3 cells treated with different microspheres in 6-well transwell chamber, the Live/Dead fluorescent staining assay (Thermo Fisher Scientific Inc., United States) was further performed to clarify the killing effects of the microspheres on tumor cells in vitro.

### The mechanism of ADM/Fe_3_O_4_-MS against HCC

Based on the presence of iron ions in the ADM/Fe_3_O_4_-MS and the previous studies [36, 37], which was the characteristics of ferroptosis-mediated cell death, we hypothesized that the ferroptosis was also involved in the molecular mechanism of ADM/Fe_3_O_4_-MS against HCC. Therefore, the specific ferroptosis inhibitors of the lipophilic antioxidant ferrostatin-1 (Fer-1, 10 μM) and the iron chelating agent deferoxamine (DFO, 150 μM) was used to test the features of ferroptosis in different microspheres treated LM3 cells. LM3 cells were also placed in a 12-well transwell chamber (Corning, Inc., Lowell, MA) with a density of 5 × 10^4^ cells per chamber and incubated overnight. Then, the MTT assay was used to detect the effect of Fer-1 or DFO intervention on cell viability.

Glutathione peroxidase 4 (GPX4) and Acyl-CoA synthetase long-chain family member 4 (ACSL4) are two key regulators of ferroptosis, and the former is a specific and robust central regulator of ferroptotic cell death, which is inactivated with the consumption or depletion of GSH [38, 39]; the latter could induce the accumulation of lipid intermediates and could also be used as an indicator of ferroptosis [40]. We further detected the expression levels of the above two proteins in LM3 cells treated with different microspheres to determine whether iron death is involved in the process of cell death.

Firstly, western blotting study was applied, the LM3 cells treated by different microspheres were collected and washed by PBS for lysis, then, the cell lysates were collected after centrifugation at 12,000 rpm and 4 °C for 10 min. After measuring the protein concentration of the total lysate using Bio-Rad DC protein assay kit (Bio-Rad, USA), the cell lysates containing loading buffer were analyzed by electrophoresis denaturing polyacrylamide gels, and the antibodies against GPX4 (Abcam, UK) and ACSL4 (Santa Cruz, CA) were used to determine the levels of the above two selected proteins. Beta-actin (β-actin; Beyotime Institute of Biotechnology, China) was used as internal reference.

Secondly, real-time quantitative PCR (RT-qPCR) was also performed, the total RNA was extracted using TRIzol reagent (Invitrogen, USA) according to the manufacturer's protocol, and the amplification primers for β-actin, GPX4 and ACSL4 were synthesized by Sangon Biotech Co., Ltd (Shanghai, China). 40 cycles of PCR were carried out at 60 ℃, and each sample was analyzed with independent procedures. After that, the relative GPX4, ACSL4 and SLC7A11 expression levels were obtained and normalized to the internal control gene β-actin.

### Creation of VX2 tumors on rabbits

The VX2 tumor was propagated by the transplanted tumor of the rabbit model, which were left based on our previous research and stored in liquid nitrogen, and the orthotopic models of liver tumors were also established as our previously reports [18, 41]. Briefly, 31 male New Zealand white rabbits (2–2.5 kg in weight) were prepared, and one was the donor rabbit and the other thirty were the intervention ones. The tumor was cut into small pieces using sterilized ophthalmic tweezers, then the minced fragments were ground to form a tumor suspension by PBS. After that, 0.2 mL of the above tumor suspension was injected subcutaneously into the hindlimb of the donor rabbit, and the rabbit was observed daily for tumor growth. After 2 weeks of the implantation, the VX2 tumor inoculated in the donor rabbit was taken out and removed the necrotic tissue, and retained the active VX2 tumor tissue and cut into 1 mm^3^ tumor fragments for further molding.

The remaining 30 rabbits were used to establish an orthotopic VX2 liver cancer model. All the rabbits were anesthetized with an intravenous injection of 2% pentobarbital sodium (1 mL/kg; Sigma-Aldrich, MO). Then, laparotomy with a 3–4 cm incision along the xiphoid process was made to expose the left hepatic lobe, and the minced tumor fragment was directly implanted into the subcapsular parenchyma of the left hepatic lobe, followed by closure of the abdominal incision with layered sutures. All tumor-bearing rabbits were scanned by magnetic resonance imaging (MRI) 2 weeks after tumor inoculation to confirm whether the modeling was successful.

All the 30 VX2 tumor-bearing rabbits were randomly divided into five groups: control group (n = 6), injections of physiological saline; blank microsphere group (MS group, n = 6), injection of gelatin microspheres; ADM-MS group (n = 6), injection of ADM-MS; ADM/Fe_3_O_4_-MS (−) group (n = 6), injection of ADM/Fe_3_O_4_-MS and without microwave intervention; ADM/Fe_3_O_4_-MS (+) group (n = 6), injection of ADM/Fe_3_O_4_-MS and with microwave intervention.

### TACE treatment procedures

TACE was carried out on 2 week after tumor implantation, and the operation process refers to our previous research [41]. Briefly, the rabbits were anesthetized by 2% pentobarbital sodium (1 mL/Kg; Sigma-Aldrich, MO). Then, a 4-F vascular arterial sheath (Cook, Bloomington, USA) was placed into the femoral artery, and then 2.7F microcatheter (Terumo, Tokyo, Japan) was superselected to the left hepatic artery under the guidance of DSA angiographic unit (AlluraXper, Philips Healthcare, Nederland), which can guarantee tumor targeted embolization. Subsequently, 100 mg/Kg of the corresponding microspheres mixing with contrast agent was carefully injected into the tumor feeding artery via hyper-selective microcatheter to ensure complete embolization of the tumor. For the ADM/Fe_3_O_4_-MS (+) group, microwave intervention (25 W) was performed 24 h after TACE for 30 min, and then performed every 2 days thereafter. The microwave probe was positioned 1 cm away from the fixed animal and oriented towards the tumor during microwave therapy.

### Evaluation of in vivo anti-tumor efficacy

To further confirm the efficacy of different microspheres in embolization therapy, MRI scans were performed 1 day before treatment and 14 days after treatment respectively, and the changes in tumor volume were determined through MRI follow-up. All the liver MRI scans of the rabbits were performed by 3.0 T Clinical MR imaging scanner (Ingenia, Philips Healthcare, Best, The Netherlands), and the T2-weighted images of liver were acquired using the following parameters: repetition time/echo time, 2,010–4,200 ms; time of echo, 43 ms; echo train length, 14; section thickness, 2 mm; field of view, 150 × 150 mm; matrix, 256 × 256; number of excitations, 2.

### Pathological staining and histological analysis

All the rabbits were sacrificed 14 days after TACE by intravenous injection of overdose of pentobarbital sodium for histopathological evaluation. The liver was removed and formaldehyde-fixed, and the paraffin-embedded tissue blocks were prepared. All the paraffin-embedded samples were stained with hematoxylin–eosin (H&E), Ki-67 immunostaining (Abcam, Cambridge, MA) and TUNEL assay (Roche, Mannheim, Germany) according to the manufacturer’s instructions. Moreover, the heart, lung, kidney and spleen were also harvested and stained with H&E for histopathological analysis. All the histopathological pictures were finally taken using the microscopy imaging system (Leica DMi8, Germany).

### Statistical analysis

Data analysis was performed using SPSS 19.0 (SPSS Inc, Chicago, IL). Quantitative data were expressed as mean ± SD. Comparisons between the groups were made by Student’s t test and one-way ANOVA. P < 0.05 was considered indicative of a statistically significant difference.

## Results and discussion

### Synthesis and characterization of MS, ADM-MS and ADM/Fe_3_O_4_-MS

In the present study, the high-voltage electrospray method was used to prepare the different microspheres. When the electrosprayed solution was pressed out by a syringe pump, a droplet of solution hung on the pinpoint of the nozzle due to surface tension. If the sum of the gravity and electrodynamic force exceeds the surface tension, the big droplet will disperse into the smaller droplets and drop into the collecting solution containing glutaraldehyde. Subsequently, the glutaraldehyde could instantly diffuse into the droplet, thereby crosslinking the gelatin droplets to form the solidified microspheres. In order to obtain microspheres that match the clinical particle size, the effects of different preparation conditions were investigated, especially the voltage, needle diameter and flow rate.

The formation of droplet is mainly the result of electrostatic force and droplet surface tension during electrospray [42, 43]. The electrostatic force causes the droplet to disperse into tiny droplets, while the surface tension does the opposite [44]. The electrostatic force of the droplet is mainly regulated by voltage. The effects of voltage on the particle size of microspheres were investigated. The results showed that the particle sizes of ADM/Fe_3_O_4_-MS were 812.3 ± 139.5, 445.3 ± 104.8 and 217.7 ± 57.4 μm when the applied voltage was 10, 15 and 20 kV, respectively (Fig. 1b). The results indicated that the particle size of microspheres decreased with the increase of voltage, which may be because the same charge accumulated in the droplet gradually increases with the increase of voltage, and therefore the repulsion caused by the same charge is greater, which could more easily disperse the droplet to the smaller ones [45, 46].

To further investigate the effect of surface tension on the preparation of ADM/Fe_3_O_4_-MS by high-voltage electrospray method, the particle sizes of the microspheres prepared by three different needle diameters were compared. The results showed that the microspheres prepared using needles with inner diameters of 190 μm, 260 μm and 510 μm were 217.7 ± 57.4 μm, 593.3 ± 103.6 μm and 849.8 ± 101.9 μm, respectively (Fig. 1c). The effect of the inner diameter of the needle on the particle size of the microspheres is mainly through the influence of the surface tension of the droplet. The results indicated that the surface tension was proportional to the surface area of the needle, and with the decrease of the inner diameter of the needle, the surface tension of the droplet decreases, resulting in smaller average particle sizes of the microspheres.

Injection flow rate also had a great influence on the particle size of microspheres, and three different flow rates (3, 6 and 10 mL/h) were set to further clarify its influence on the particle size of microspheres. The results showed that the particle sizes of the prepared microspheres for 3, 6 and 10 mL/h were 217.7 ± 57.4 μm, 434.7 ± 93.2 μm and 717.5 ± 90.0 μm, respectively (Fig. 1d). The research results indicate that the particle size of the microspheres increases with the increase of the injection flow rate of the syringe, which was due to that with the increase of the injection flow rate, the droplets that fall in a unit time tend to be larger, and the particle size of the microspheres formed also increased.

To prepare microspheres with particle size matching the clinical TACE treatment process of liver cancer, based on the influence of the above different parameters on the particle size of microspheres, the selected voltage was 20 kV, the inner diameter of the needle was 190 μm, and the injection flow rate was 3 mL/h. Meanwhile, 2% of gelatin and 1% of glutaraldehyde were also applied. According to the above conditions, MS, ADM-MS and ADM/Fe_3_O_4_-MS with relatively uniform particle sizes were prepared, which were 223.3 ± 91.1 μm, 210.8 ± 71.8 μm and 217.7 ± 57.4 μm, respectively (Additional file 1: Fig. S1). The particle sizes of the most microspheres were between 200 and 300 μm, and the shape of the microspheres remained spheroidal (Fig. 1e).

### Drug loading capacity and encapsulation efficiency of ADM-MS and ADM/Fe_3_O_4_-MS

**Fig. 2:**
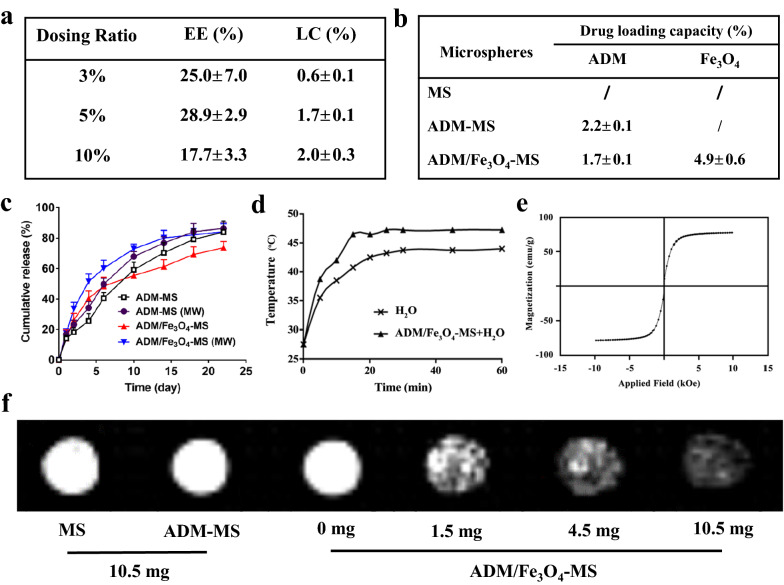
Physicochemical properties of different drug-loaded microspheres. **a** The influence of the dosing ratio on drug loading capacity (LC) and encapsulation efficiency (EE) of ADM/Fe_3_O_4_-MS. **b** The drug loading capacity of different microspheres. **c** In vitro cumulative release curve of adriamycin in ADM-MS and ADM/Fe_3_O_4_-MS with or without microwave intervention irradiation. **d** Heating curve of the aqueous solution containing ADM/Fe_3_O_4_-MS heated by microwave. **e** Hysteresis curves of ADM/Fe_3_O_4_-MS. **f** In vitro T2-weighted images of MS, ADM-MS and ADM/Fe_3_O_4_-MS with different contents

The drug loading capacity (LC) and encapsulation efficiency (EE) of the microspheres are also two important performance indicators of the drug-loaded microspheres, which could directly affect the TACE treatment effect of HCC [47, 48], and were further determined in the present study. We first determined the influence of the ADM dosing ratio (3–10%) on the LC and EE of ADM/Fe_3_O_4_-MS. As shown in Fig. 2a, when the dosing ratio was 3%, the LC and EE of the prepared ADM/Fe_3_O_4_-MS were 0.6 ± 0.1% and 25.0 ± 7.0%, respectively; when the dosing ratio was 5%, LC and EE were 1.7 ± 0.1% and 28.9 ± 2.9%, respectively; when the dosing ratio was 10%, LC and EE were 2.0 ± 0.3% and 17.7 ± 3.3%, respectively. The above results suggested that LC gradually increased with the increase of dosing ratio, but the increasing trend decreased significantly when dosing ratio reached 5%. Moreover, we also found that EE was optimal when the dosage ratio was 5%, but decreased significantly when it reached 10%. Based on the above results, we can speculate that in a range of dosing ratio, the LC was greatly increased when the dosing ratio increased, while the EE of ADM/Fe_3_O_4_-MS was reduced. In our present study, the optimized dosing ratio for ADM/Fe_3_O_4_-MS was 5%, which was further selected for the preparation of ADM/Fe_3_O_4_-MS.

In this study, the LC of the prepared microspheres was further confirmed. The results of the study showed that when the dosing ratio was 5%, the LC of adriamycin in ADM-MS was 2.2 ± 0.1%, while the loading capacity of adriamycin in ADM/Fe_3_O_4_-MS was 1.7 ± 0.1%, and the LC of Fe_3_O_4_ nanoparticles is 4.9 ± 0.6% (Fig. 2b). The results indicated that both ADM-MS and ADM/Fe_3_O_4_-MS could effectively encapsulate adriamycin, but the LC of ADM/Fe_3_O_4_-MS was slightly lower than ADM-MS, which may be due to the simultaneous loading of Fe_3_O_4_ nanoparticles affect the encapsulation of adriamycin within microspheres to a certain extent.

### In vitro drug release behavior of ADM-MS and ADM/Fe_3_O_4_-MS

As drug-loaded microspheres, we have further investigated the in vitro drug release behavior of ADM-MS and ADM/Fe_3_O_4_-MS. The cumulative release percentages of adriamycin from ADM-MS and ADM/Fe_3_O_4_-MS were observed, and we found that both of ADM-MS and ADM/Fe_3_O_4_-MS were released in a biphasic pattern, with more than 70% of the adriamycin was released from the microspheres within 22 days (Fig. 2c). For ADM/Fe_3_O_4_-MS, it showed a rapid release of adriamycin in the initial 10 days, and its cumulative release percentage was exceeded 50%, followed by a sustained release up to 73.8 ± 4.0% after 22 days. Compared to ADM/Fe_3_O_4_-MS, the adriamycin release percentage of ADM-MS was lower in the initial 10 days and the final cumulative drug release amount was 84.0 ± 7.3% after 22 days. ADM/Fe_3_O_4_-MS and ADM-MS showed different adriamycin release behaviors, especially ADM/Fe_3_O_4_-MS showed a rapid drug release in the first 10 days, which may due to the fact that the voids caused by the release of Fe_3_O_4_ nanoparticles from ADM/Fe_3_O_4_-MS was beneficial for the release of adriamycin. The above results also suggested that ADM/Fe_3_O_4_-MS and ADM-MS could slowly release the encapsulated drug, thereby reducing the incidence of side effects and promoting the efficacy of chemotherapy.

Meanwhile, we further clarified the influence of microwave on drug release behavior of ADM/Fe_3_O_4_-MS and ADM-MS. For ADM-MS, we found that ADM-MS showed a small increase in drug release at each time point under microwave irradiation, and at 22 days, the cumulative drug release amount for the ADM-MS plus microwave intervention was reached to 86.3 ± 4.1%, compared with that without microwave intervention, there was no statistical difference. Compared with ADM-MS, we found that microwave irradiation had a greater effect on drug release behavior of ADM/Fe_3_O_4_-MS, significantly increasing drug release at each time point, up to 84.2 ± 5.1% at day 22. The above results also suggested that microspheres loaded with Fe_3_O_4_ nanoparticles can significantly increase the release amount of chemotherapy drugs under the action of microwave, which has a positive effect on improving the anti-tumor efficacy of microspheres.

### Thermal efficiency of ADM/Fe_3_O_4_-MS

The thermal efficiency of ADM/Fe_3_O_4_-MS when heated by microwave was also investigated in our present study. The temperature change of the aqueous solution containing ADM/Fe_3_O_4_-MS heated by microwave at different times was recorded by a thermometer, and the results showed that compared with the aqueous solution without ADM/Fe_3_O_4_-MS, the aqueous solution containing ADM/Fe_3_O_4_-MS heated by microwaves had a faster temperature rise and a larger temperature rise range at the same time (Fig. 2d). The temperature of the aqueous solution in the presence of ADM/Fe_3_O_4_-MS increased from 27.5 to 47.5 °C, and the temperature of the aqueous solution without ADM/Fe_3_O_4_-MS only rise from 27.5 to 44.0 °C in 60 min. In the first 15 min, the temperature of the aqueous solution containing ADM/Fe_3_O_4_-MS increased by 20 °C, while the control group only increased by 12.5 °C. The results suggested that Fe_3_O_4_ nanoparticles could endow ADM/Fe_3_O_4_-MS with a higher thermal efficiency, and it is feasible to stimulate the heat generation of the microspheres by microwave [23, 24]. Meanwhile, it also provided an important basis for this study to use the microwave-mediated thermal energy of ADM/Fe_3_O_4_-MS to improve the anti-tumor efficacy.

### MRI performance and superparamagnetism of ADM/Fe_3_O_4_-MS

Due to the introduction of Fe_3_O_4_ nanoparticles, a negative contrast agent [49], ADM/Fe_3_O_4_-MS is not only a drug-loaded microsphere, but also a magnetic microsphere, giving the microspheres with visibility under MRI. The in vitro MRI performance of ADM/Fe_3_O_4_-MS was also confirmed in our present study, and we performed T2-weighted imaging scans of 0, 1.5, 4.5, 10.5 mg ADM/Fe_3_O_4_-MS, 10.5 mg MS and ADM-MS (Fig. 2f). The results showed that with the quality of ADM/Fe_3_O_4_-MS increased, the T2-weighted image gradually darkened. In addition, there was no significant change in T2 signals of MS and ADM-MS, which were far inferior to ADM/Fe_3_O_4_-MS with same weight. Furthermore, the T2-weighted images of ADM/Fe_3_O_4_-MS were significantly darker than those of MS and ADM-MS, indicating that ADM/Fe_3_O_4_-MS had good magnetic resonance imaging effect. Correspondingly, we also detected the T2-weighted relaxation value corresponding to Fig. 2f (Additional file 1: Fig. S2), and the results showed that T2-weighted relaxation value was decreased with increasing Fe concentration, and 10.5 mg ADM/Fe_3_O_4_-MS was the lowest among all the groups.

The superparamagnetism of ADM/Fe_3_O_4_-MS was also confirmed, and Fig. 2e showed the hysteresis curve. The results showed that the magnetization intensity of ADM/Fe_3_O_4_-MS increased obviously with the increase of the intensity of external magnetic field. On the one hand, when the intensity of the magnetic field increased to a certain extent, the magnetization of ADM/Fe_3_O_4_-MS reached the maximum and keep steady. One the other hand, when the external magnetic field was removed, the remanent magnetization was changed to zero. The above results indicated that ADM/Fe_3_O_4_-MS had superparamagnetism, and the high-voltage electrospraying method could prepare ADM/Fe_3_O_4_-MS without destroying the properties of Fe_3_O_4_ nanoparticles.

### Cytotoxicity and inhibitory effect of MS, ADM-MS and ADM/Fe_3_O_4_-MS

**Fig. 3:**
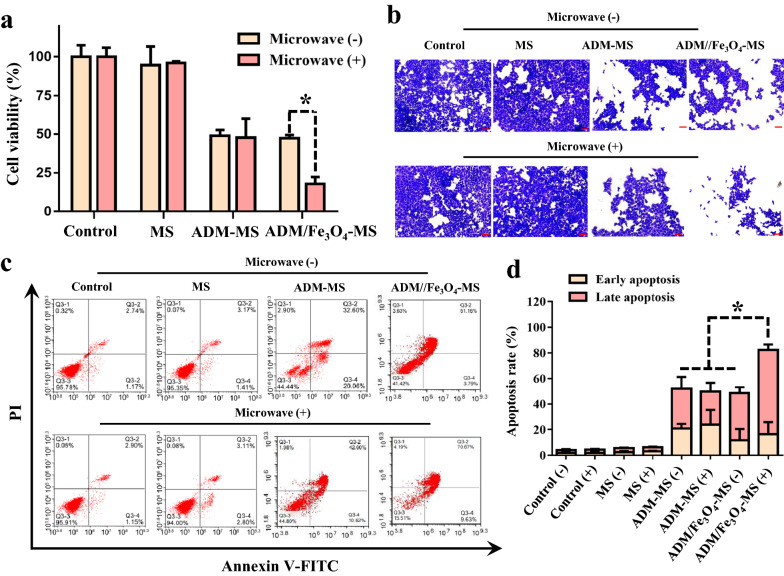
Cytotoxicity and in vitro inhibitory effect of MS, ADM-MS and ADM/Fe_3_O_4_-MS. **a** Effect of co-incubation of MS, ADM-MS and ADM/Fe_3_O_4_-MS with LM3 cells with or without microwave intervention on their cell viability by MTT assay. **b** Effects of MS, ADM-MS and ADM/Fe_3_O_4_-MS on cell proliferation ability with or without microwave by cell clonogenesis assay and corresponding crystal violet staining. The scale bar is 100 μm. **c** Effects of MS, ADM-MS and ADM/Fe3O4-MS on LM3 cell apoptosis with or without microwave by Annexin V−PI apoptosis detection. **d** The results of quantitative analysis of apoptotic cells based on Annexin V−PI apoptosis detection

To determine the cytotoxicity of MS, ADM-MS and ADM/Fe_3_O_4_-MS, we explored their killing effect on tumor cells with or without microwave intervention. The cells without microsphere treatment was serves as the control group. The MTT assay results showed that without microwave intervention, the cell viability of MS group, ADM-MS group and ADM/Fe_3_O_4_-MS group were 94.5 ± 12.1%, 48.9 ± 3.8% and 47.3 ± 2.2%, respectively; after microwave intervention, the cell viability was changed to 95.9 ± 1.1%, 47.6 ± 12.6% and 17.9 ± 4.4%, respectively (Fig. 3a). The results showed that the presence or absence of microwave exposure has no significant effect on cell viability for MS and ADM-MS. Interestingly, for ADM/Fe_3_O_4_-MS, we found that the cell viability was significantly reduced after microwave intervention, suggesting that the introduction of Fe_3_O_4_ nanoparticles into ADM/Fe_3_O_4_-MS could enhance the antitumor effect under microwave-mediated hyperthermia. Previous studies have confirmed that hyperthermia could trigger drug release from different drug delivery system and further improve the accumulation, distribution, and efficacy of chemotherapeutic drugs [26, 50], which explained why ADM/Fe_3_O_4_-MS could enhance the tumor killing effect under microwave irradiation.

The clonogenic assay was also used to detect the changes of cell proliferation ability after different microspheres treatments in the present study. Crystal violet staining showed that microwave intervention had no significant effect on cell proliferation in the control, MS, and ADM-MS group (Fig. 3b). Meanwhile, we also found that the number of cells in the ADM/Fe_3_O_4_-MS group after microwave treatment was significantly reduced, which was significantly less than that of the group without microwave exposure, suggesting that the proliferation ability of cells after microwave treatment was significantly weakened, which was also consistent with the above MTT results.

Annexin V-PI staining was used to further investigate the effect of MS, ADM-MS and ADM/Fe_3_O_4_-MS on the apoptosis of LM3 hepatoma cells with or without microwave treatment. Flow cytometry analysis showed that without microwave treatment, the proportion of early apoptotic cells in the control, MS, ADM-MS and ADM/Fe_3_O_4_-MS group was 1.79 ± 0.55%, 2.4 ± 0.9%, 20.8 ± 3.7% and 11.6 ± 8.8%, respectively; the proportion of late apoptotic cells was 2.2 ± 0.8%, 3.1 ± 0.4%, 31.2 ± 9.3% and 36.8 ± 4.7%, respectively (Fig. 3c). After microwave treatment, the proportion of early apoptotic cells in the control, MS, ADM-MS and ADM/Fe_3_O_4_-MS group was changed to 1.8 ± 0.8%, 3.0 ± 0.5%, 23.7 ± 11.6% and 16.3 ± 9.5%, respectively; the proportion of late apoptotic cells was changed to 2.5 ± 0.6%, 3.2 ± 0.5%, 26.1 ± 6.8% and 66.0 ± 4.2%, respectively (Fig. 3c). The results also showed that microwave treatment had no significant effect on apoptosis in the control, MS and ADM-MS group, and there was no significant difference between the group without microwave treatment. For ADM/Fe_3_O_4_-MS, we also observed that the total apoptosis ratio of ADM/Fe_3_O_4_-MS was significantly increased after microwave treatment, which was significantly higher than that of the other groups (Fig. 3d), suggesting that microwave-induced hyperthermia could enhance the tumor killing effect of ADM/Fe_3_O_4_-MS.

**Fig. 4:**
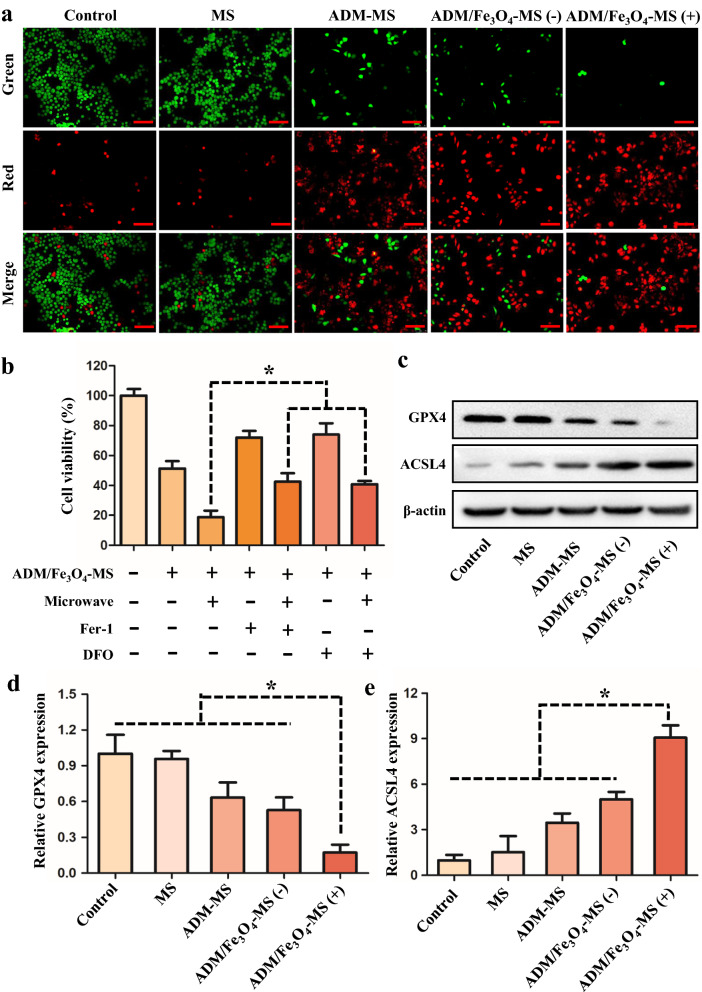
In vitro anti-tumor efficacy of MS, ADM-MS and ADM/Fe_3_O_4_-MS and the evaluation of ferroptosis. **a** Live/dead cell staining of MS, ADM-MS and ADM/Fe3O4-MS after co-incubation with LM3 cells. The live cells appeared green, whereas the dead cells appeared red. The scale bar is 100 μm. **b** Cell viability after treated with Fer-1 (10 μM) and DFO (150 μM) and ADM/Fe_3_O_4_-MS with or without microwave irradiation. **c** Western blot analysis of GPX4 and ACSL4 expression in LM3 cells after the treatment with different formulas. **d**, **e** RT-qPCR analysis of GPX4 and ACSL4 gene expression in LM3 cells after the treatment with different formulas

Live/Dead fluorescent staining assay was further used to determine the cytotoxicity of different microspheres in tumor cells, especially the killing effect of ADM/Fe_3_O_4_-MS under microwave intervention. As shown in Fig. 4a, we found that there was no significant difference between the cell survival of MS group and the control group, which suggested that the wall material of microspheres in our study was low or non-toxic. Meanwhile, we also found that the cytotoxicity of ADM/Fe_3_O_4_-MS was similar to that of ADM-MS in the absence of microwave intervention, suggesting that loading Fe_3_O_4_ nanoparticles did not enhance the antitumor effect of the microspheres. After microwave intervention, we found that the tumor cell killing effect of ADM/Fe_3_O_4_-MS was significantly enhanced, which was superior to that of ADM/Fe_3_O_4_-MS alone. The above results further confirmed our assumption that ADM/Fe_3_O_4_-MS combined with microwave could enhance anti-tumor efficiency.

### Mechanistic evaluation of ferroptosis induced by ADM/Fe_3_O_4_-MS

Since Fe_3_O_4_ nanoparticles were introduced into the microsphere system, we hypothesized that ferroptosis was also involved in the cell death process, which was a type of regulated cell death driven by the iron-dependent accumulation [51, 52]. Therefore, two specific inhibitors (Fer-1 and DFO) were applied to verify the features of ferroptosis in ADM/Fe_3_O_4_-MS treated LM3 cells. Ferrostatin-1 (Fer-1) was the lipophilic radical scavenger, which had been identified as an effective inhibitor of ferroptosis [53]. Deferoxamine (DFO) was the iron chelator, which could inhibit the ROS accumulation and lipid peroxidation to suppress ferroptosis [54]. As shown in Fig. 4b, we could find that the two ferroptosis inhibitors Fer-1 and DFO both could reduce the cytotoxicity caused by ADM/Fe_3_O_4_-MS plus microwave irradiation, and the cell activity was significantly higher than that of the group without inhibitors, suggesting that ferroptosis was indeed involved in the process of cell death. Moreover, the reversal of the cell death by ferroptosis inhibitors and iron-chelating agents further confirmed our hypothesis.

To further confirm the ferroptosis was involved in the anti-tumor effect of ADM/Fe_3_O_4_-MS under microwave irradiation, the changes of GPX4 and ACSL4, two biomarkers of ferroptosis, were analyzed. GPX4 was the lipid hydroperoxidase that could convert lipid hydroperoxides to lipid alcohols, which prevented the iron-dependent formation of toxic lipid reactive oxygen species (ROS) [55]. What’s more, the function of GPX4 was preventing oxidative lipid damage, when the function of GPX4 was inhibited, it could lead to lipid peroxidation and cause ferroptosis, so it was one of the key markers of ferroptosis [38]. ACSL4 was a member of the long chain family of acyl‑CoA synthetase proteins, which not only could promote the formation of phytosterol esters esterified from arachidonic acid (AA) and adrenaline, but also could drive ferroptosis via the accumulation of oxidized cellular membrane phospholipids, which was also one of the important markers for ferroptosis [56, 57]. In the present study, we evaluated the protein levels of GPX4 and ACSL4 after treatment with different microspheres. As shown in Fig. 4c, the protein levels of GPX4 was slightly downregulated when LM3 cells was treated with ADM/Fe_3_O_4_-MS compared with the other groups, indicating the occurrence of ferroptosis. Interestingly, GPX4 expression was further reduced with microwave intervention compared to without microwave, suggesting that microwave mediated hyperthermia could further enhance ferroptosis induced by ADM/Fe_3_O_4_-MS. Moreover, the protein levels of ACSL4 were obviously upregulated in LM3 cells treated with ADM/Fe_3_O_4_-MS compared with the other groups. In addition, the protein levels of ACSL4 were highest in LM3 cells treated with ADM/Fe_3_O_4_-MS plus microwave irradiation among all formulas, which also indicated that ferroptosis could be boosted by microwave (Fig. 4c). The results of the gene assay for GPX4 (Fig. 4d) and ACSL4 (Fig. 4e) also supported the conclusion that the ADM/Fe_3_O_4_-MS could induce ferroptosis, which could be enhanced by microwave in LM3 cells.

### In vivo antitumor efficacy of different microspheres

**Fig. 5:**
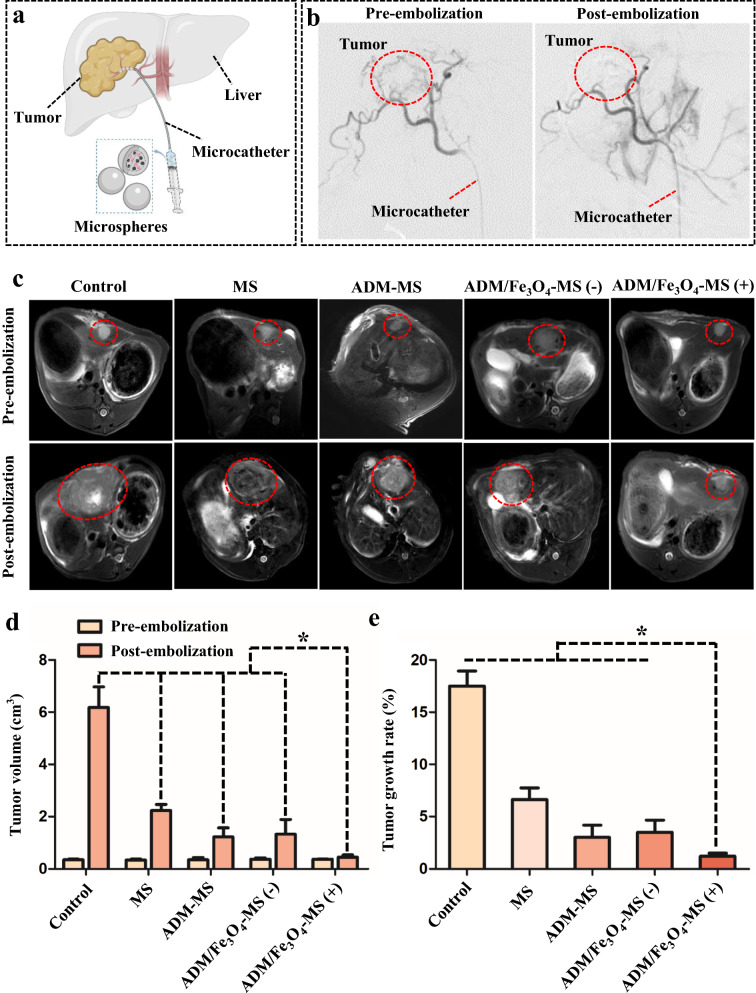
TACE treatment of the rabbit VX2 orthotopic liver cancer model and its efficacy. **a** Schematic diagram of TACE treatment process. **b** All tumor-bearing rabbits were treated with standardized TACE using different microspheres under the guidance of DSA. **c** T2-weighted imaging of tumor-bearing rabbits in the control, MS, ADM-MS, ADM/Fe_3_O_4_-MS (−) and ADM/Fe_3_O_4_-MS (+) groups before and 2 week after treatment. **d** Tumor volume of the control, MS, ADM-MS, ADM/Fe_3_O_4_-MS (−) and ADM/Fe_3_O_4_-MS (+) groups before and 2 week after treatment. **e** Tumor growth rates of the control, MS, ADM-MS, ADM/Fe_3_O_4_-MS (−) and ADM/Fe_3_O_4_-MS (+) groups

The Rabbit VX2 hepatic tumor model was used to evaluate the in vivo antitumor activity of MS, ADM-MS and ADM/Fe_3_O_4_-MS. All rabbits confirmed to be successfully modeled were randomly assigned to each group, and then the corresponding microspheres were injected into the tumor site under the guidance of the microcatheter (Fig. 5a). In order to determine whether the tumor blood supply artery was completely blocked by the microspheres, tumor angiography with DSA before and after embolization was performed. All TACE procedures were performed by two interventional specialists with more than 10 years of clinical experience to ensure the standardization and reliability of the procedures. The result showed that the tumor blood vessels were completely blocked after the corresponding microsphere injection (Fig. 5b). Our results also showed that the MS, ADM-MS and ADM/Fe_3_O_4_-MS prepared in this study could completely embolize tumor vessels and blocked the tumor arterial blood supply as embolic materials.

All tumor-bearing rabbits in the group were followed up with MRI before TACE and 2 weeks after TACE treatment with MS, ADM-MS and ADM/Fe_3_O_4_-MS, and the rabbits without embolization treatment were applied as a control group. To clarify the therapeutic efficacy of different microspheres, the tumor volume and tumor growth rate were recorded based on the corresponding T2-weighted imaging. As shown in Fig. 5c, the representative T2-weighted imaging showed the significant changes in tumor size of different groups. Notably, we did not observe a significantly reduced signal change in T2-weighted imaging of ADM/Fe_3_O_4_-MS groups after embolization. This might be due to the fact that the tumor arteries of rabbits were much smaller compared with human tumor vessels. In this study, only a small number of microspheres were injected, and the microspheres were scattered in the tumor, which further led to no obvious signal changes in vivo.

For the changes in tumor volume, compared with the control group (tumor volume increased from 0.35 ± 0.03 cm^3^ before intervention to 6.18 ± 0.79 cm^3^ 1 week later), and tumor volume changes were significantly smaller after different microsphere embolization treatments (Fig. 5d). Among them, the tumor volume in the MS group changed from 0.34 ± 0.04 cm^3^ before TACE treatment to 2.23 ± 0.23 cm^3^ one week after TACE, and the ADM-MS group changed from 0.35 ± 0.09 to 1.22 ± 0.35 cm^3^. Moreover, the tumor volume in the ADM/Fe_3_O_4_-MS group changed from 0.37 ± 0.05 to 1.33 ± 0.56 cm^3^ without microwave intervention, and from 0.37 ± 0.01 to 0.45 ± 0.09 cm^3^ with microwave intervention. It can be found that ADM/Fe_3_O_4_-MS plus microwave irradiation could effectively inhibit tumor growth, presenting that the tumor volume was significantly smaller than the other groups. We further analyzed the tumor growth rate, which were17.5 ± 1.44%, 6.63 ± 1.12%, 3.03 ± 1.14%, 3.50 ± 1.15% and 1.20 ± 0.30% for the control, MS, ADM-MS, ADM/Fe_3_O_4_-MS (−) and ADM/Fe_3_O_4_-MS (+) groups, respectively (Fig. 5e). It was found that under the action of microwave, the therapeutic efficacy of ADM/Fe_3_O_4_-MS was significantly improved, effectively controlling the trend of tumor proliferation, and the therapeutic efficacy was significantly better than that of the other groups.

**Fig. 6:**
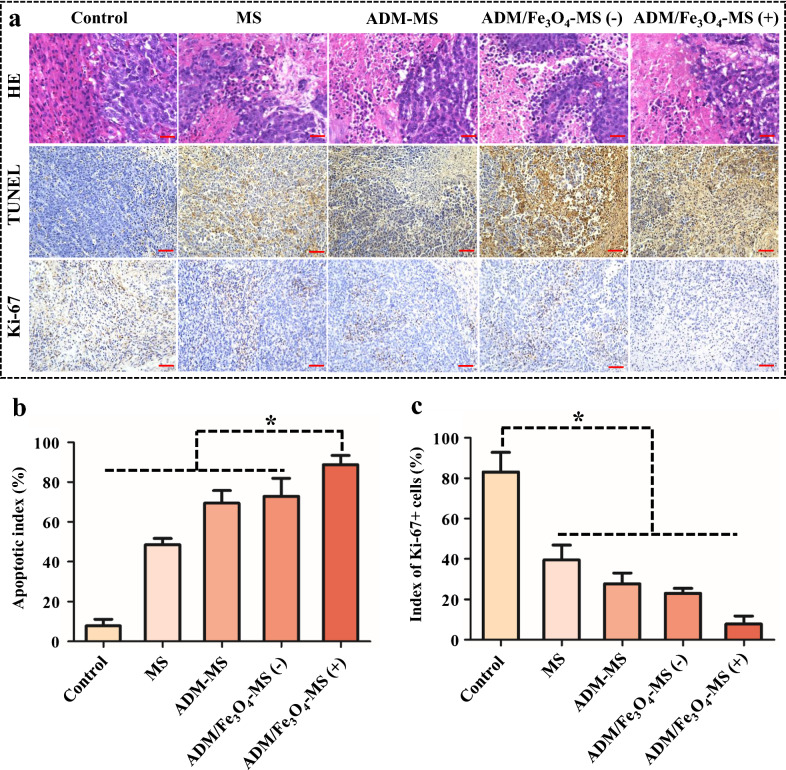
Histological analysis of tumors in tumor-bearing rabbits 2 week after treatment. **a** H&E staining, TUNEL immunohistochemical staining and Ki-67 immunostaining of tumor tissues (all tissues: 200×). The brown areas indicate TUNEL-positive and Ki-67-positive cells. **b** Index of TUNEL-positive cells in each group. **c** Index of Ki-67-positive cells in each group. Data are presented as the mean ± SD (n = 6) (*p < 0.05)

We further used histopathological staining to confirm the therapeutic effect of different microspheres for HCC (Fig. 6a). H&E staining was used to observe the tumor tissue morphology [58], and hepatocellular apoptosis were tested by TUNEL staining [59], and Ki-67 immunohistochemical staining was used to detect the changes in tumor cell proliferation after treatment [60]. The results of H&E staining showed that no obvious tumor necrosis area was observed in the control group, but it could be observed in the other groups, especially the ADM/Fe_3_O_4_-MS plus microwave irradiation group, the tumor necrosis area was significantly larger than the other groups (Fig. 6a). Correspondingly, TUNEL staining results showed that the number of tumor cells apoptosis significantly increased after different microsphere intervention, compared with the control group (7.8 ± 3.4%), the apoptotic index of MS, ADM-MS and ADM/Fe_3_O_4_-MS group reached to 48.5 ± 3.2%, 69.5 ± 6.3% and 72.8 ± 9.0%, respectively (Fig. 6b). Under the action of microwave, the tumor killing effect of ADM/Fe_3_O_4_-MS was further improved, and the proportion of apoptotic cells reached to 88.8 ± 4.7%, which was significantly higher than the other groups (Fig. 6b). On the other hand, the results of Ki-67 immunohistochemical staining also showed that compared with the control group (83.1 ± 9.8%), Ki-67-positive cells were reduced to 39.6 ± 7.3%, 27.7 ± 5.3% and 22.9 ± 2.5% after MS, ADM-MS and ADM/Fe_3_O_4_-MS intervention, respectively (Fig. 6c). When ADM/Fe_3_O_4_-MS was combined with microwave, the Ki-67-positive cells were further reduced to 7.9 ± 3.9%, which was significantly lower than the other groups (Fig. 6c). The above results indicated that the ADM/Fe_3_O_4_-MS had a superior anti-tumor efficiency compared with the other microspheres, and its tumor killing effect could be further improved under the action of microwave.

### Biosafety of MS, ADM-MS and ADM/Fe_3_O_4_-MS

**Fig. 7:**
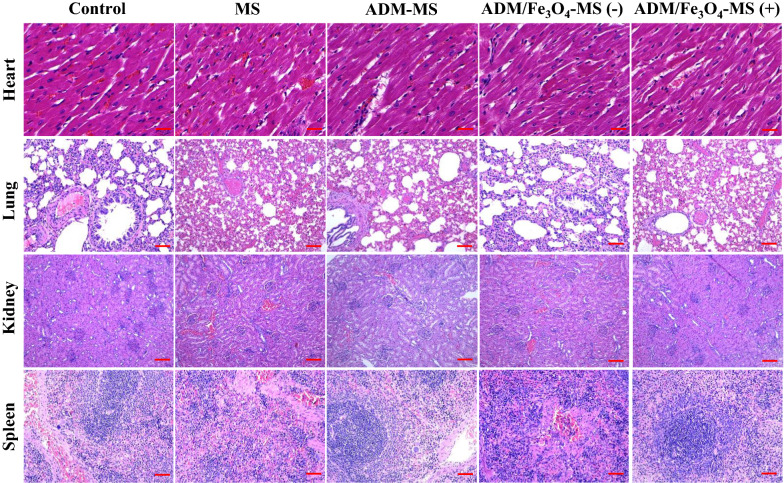
Representative major organ tissue (heart, lung, kidney and spleen) stained with H&E 2 week after treatment. Scale bar represents 100 μm

Histopathological analysis was also applied to determine the biosafety of different microspheres and their effects on the major organs (heart, lung, kidney and spleen) of rabbits (Fig. 7). Cardiomyopathy was the most dangerous adverse effect of adriamycin [61], and no apparent signs of heart failure caused by MS, ADM-MS and ADM/Fe_3_O_4_-MS were observed, including disordered myocardial fiber arrangement, myocardial fiber rupture and mitochondria damage, which suggested that the encapsulation of adriamycin through microspheres could not only effectively exert its tumor cell killing effect, but also can effectively alleviate or even eliminate its cardiotoxicity. Meanwhile, we also found that the lung, kidney and spleen of rabbits after the intervention of MS, ADM-MS and ADM/Fe_3_O_4_-MS did not have obvious pathological changes, further confirming their biological safety. The biosafety evaluation of different microspheres also provides important support for the potential clinical application of microspheres prepared in the present study.

## Conclusion

Overall, a novel microsphere co-loaded with Fe_3_O_4_ nanoparticles and adriamycinwas was constructed for TACE treatment in this study, and the Fe_3_O_4_ nanoparticles were introduced into the chemoembolization system to improve the anti-HCC efficacy. The ADM/Fe_3_O_4_-MS could be visible under MRI and generate heat under microwave stimulation due to the effective loading ability of Fe_3_O_4_ nanoparticles. The tumor killing ability of ADM/Fe_3_O_4_-MS could be significantly improved under microwave irradiation. We further found that ferroptosis was involved in the process of tumor cell death, in which ferroptosis marker GPX4 was significantly decreased and ACSL4 was significantly increased. Meanwhile, ferroptosis inhibitors could reverse this killing effect caused by ADM/Fe_3_O_4_-MS plus microwave irradiation. In vivo study based on rabbit VX2 hepatic tumor model was also confirmed that ADM/Fe_3_O_4_-MS plus microwave irradiation can achieve superior therapeutic effects with few abnormalities. These satisfactory results suggested that Fe_3_O_4_ nanoparticles could significantly improve the overall therapeutic efficacy of drug-loaded microspheres without affecting the effective entrapment of chemotherapeutic drugs, which was a microsphere type with broad prospects for clinical transformation, especially in tumor chemoembolization.

## Supplementary Information

**Additional file 1: Figure S1.** The particle size of MS, ADM-MS and ADM/Fe_3_O_4_-MS. The results showed that the prepared MS, ADM-MS and ADM/Fe_3_O_4_-MS in this study had relatively uniform particle sizes, and the particle sizes of most microspheres were between 200 to 300 μm, which were 223.3 ± 91.1 μm, 210.8 ± 71.8 μm and 217.7 ± 57.4 μm, respectively. **Figure S2.** T2-weighted relaxation value of MS, ADM-MS and ADM/Fe_3_O_4_-MS with different contents.

## Data Availability

The datasets used and analyzed during the current study are available from the corresponding author on reasonable request.
