# Supplementary material for: Homogenous multifunctional microspheres induce ferroptosis to promote the anti-hepatocarcinoma effect of chemoembolization
Source: J Nanobiotechnology. 2022 Apr 2;20:179. doi: 10.1186/s12951-022-01385-x (PMC8976998; doi:10.1186/s12951-022-01385-x)
Supplement: Supplementary file 1 — Additional file 1: Figure S1. The particle size of MS, ADM-MS and ADM/Fe3O4-MS. The results showed that the prepared MS, ADM-MS and ADM/Fe3O4-MS in this study had relatively uniform particle sizes, and the particle sizes of most microspheres were between 200 to 300 μm, which were 223.3 ± 91.1 μm, 210.8 ± 71.8 μm and 217.7 ± 57.4 μm, respectively. Figure S2. T2-weighted relaxation value of MS, ADM-MS and ADM/Fe3O4-MS with different contents. [file 12951_2022_1385_MOESM1_ESM.docx]

**Supporting Information**

**Homogenous multifunctional microspheres induce ferroptosis to promote the anti-hepatocarcinoma effect of chemoembolization**

Minjiang Chen^1,2†^, Jie Li^2, 3†^, Gaofeng Shu^2†^, Lin Shen^2^, Enqi Qiao^2^, Nannan Zhang^2^, Shiji Fang^2^, Xiaoxiao Chen^2^, Zhongwei Zhao^2^, Jianfei Tu^2^, Jingjing Song ^2*^, Yongzhong Du^1*^, Jiansong Ji^2*^

^1^Institute of Pharmaceutics, College of Pharmaceutical Sciences, Zhejiang University, Hangzhou, 310058, China.

^2^Key Laboratory of Imaging Diagnosis and Minimally Invasive Intervention Research, Lishui Hospital of Zhejiang University, the Fifth Affiliated Hospital of Wenzhou Medical University, Lishui, 323000, China.

^3^Department of Medical Imaging, Ningbo Women & Childen's Hospital, Ningbo, 315012, China

^†^ Minjiang Chen, Jie Li and Gaofeng Shu contributed equally to this work.

***Correspondence to:**

**Jiansong Ji**, MD, PhD, Key Laboratory of Imaging Diagnosis and Minimally Invasive Intervention Research, Lishui Hospital of Zhejiang University, Lishui, 323000, China. Tel.: +86 578 2285011. E-mail: jijiansong@zju.edu.cn

**Yongzhong Du**, PhD, Institute of Pharmaceutics, College of Pharmaceutical Sciences, Zhejiang University, Hangzhou, 310058, China. Tel.: +86 571 88208435. E-mail: duyongzhong@zju.edu.cn

**Jingjing Song**, MD, Key Laboratory of Imaging Diagnosis and Minimally Invasive Intervention Research, Lishui Hospital of Zhejiang University, Lishui, 323000, China. Tel.: +86 578 2285011. E-mail: 8808s_j996@163.com


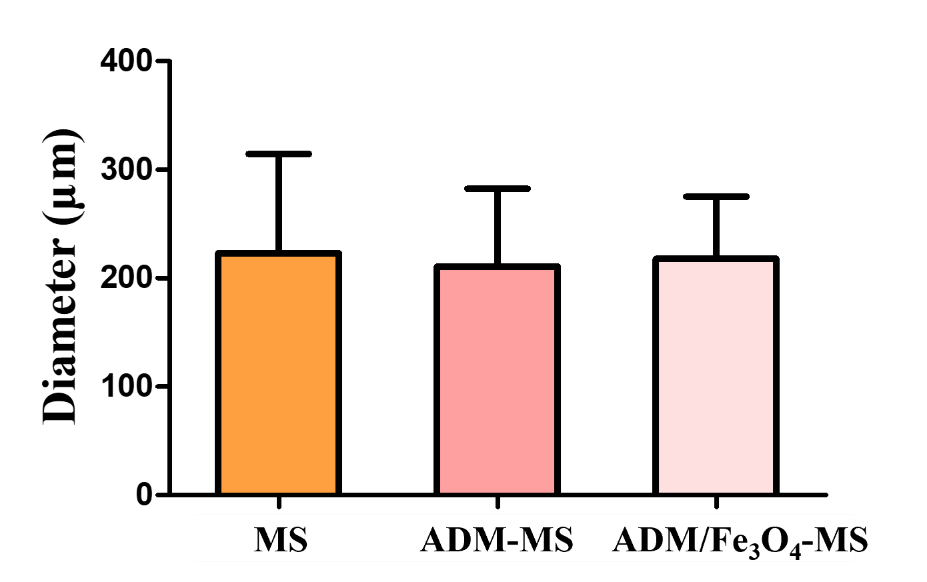


**Figure S1.** The particle size of MS, ADM-MS and ADM/Fe_3_O_4_-MS. The results showed that the prepared MS, ADM-MS and ADM/Fe_3_O_4_-MS in this study had relatively uniform particle sizes, and the particle sizes of most microspheres were between 200 to 300 μm, which were 223.3±91.1 μm, 210.8±71.8 μm and 217.7±57.4 μm, respectively.


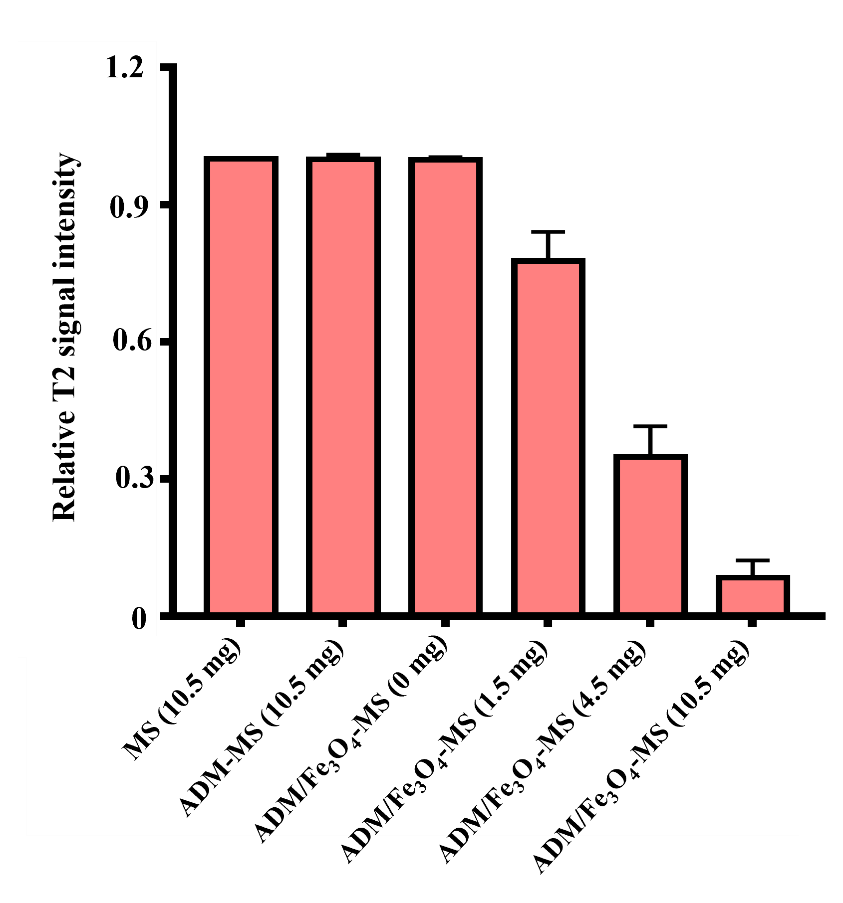


**Figure S2.** T2-weighted relaxation value of MS, ADM-MS and ADM/Fe_3_O_4_-MS with different contents.
